# The Effect of Mental Fatigue on Cognitive and Aerobic Performance in Adolescent Active Endurance Athletes: Insights from a Randomized Counterbalanced, Cross-Over Trial

**DOI:** 10.3390/jcm7120510

**Published:** 2018-12-03

**Authors:** Maamer Slimani, Hela Znazen, Nicola Luigi Bragazzi, Mohamed Sami Zguira, David Tod

**Affiliations:** 1 Postgraduate School of Public Health, Department of Health Sciences (DISSAL), Genoa University, Genoa 16132, Italy; maamer2011@hotmail.fr; 2 Department of Neuroscience, Rehabilitation, Ophthalmology, Genetics, Maternal and Child Health (DINOGMI), Section of Psychiatry, Genoa University, Genoa 16132, Italy; 3 Department of Physical Education and Sport, Faculty of Education, Taif University, Taif 21944, Saudi Arabia; znazen.hela@laposte.net; 4 Higher Institute of Sport and Physical Education of Gafsa, Gafsa 2100, Tunisia; sami-zguira@hotmail.fr; 5 School of Sport and Exercise Sciences, Liverpool John Moores University, Liverpool L3 3AF, UK; d.a.tod@ljmu.ac.uk

**Keywords:** mental exertion, RPE, aerobic performance, active endurance athletes, psychophysiology

## Abstract

The aim of this randomized counterbalanced, 2 × 2 cross-over study was to investigate the effects of mental fatigue on cognitive and aerobic performance in adolescent active endurance athletes. Ten active male endurance athletes (age = 16 ± 1.05 years, height = 1.62 ± 0.04 m, body mass = 55.5 ± 4.2 kg) were familiarized to all experimental procedures on day 1. On days 2 and 3, participants provided a rating of mental fatigue before and after completing a 30 min Stroop test that measures selective attention capacity and skills and their processing speed ability (mentally fatigued condition), or a 30 min control condition in a randomized counterbalanced order. They then performed d2 test and a 20 m multistage fitness test (MSFT), which was used to measure selective and sustained attention and visual scanning speed (i.e., concentration performance (CP) and total number of errors (E)) and aerobic fitness (i.e., maximum oxygen uptake (VO_2_max) and velocity at which VO_2_max occurs (vVO_2_max)), respectively. Rating of perceived exertion (RPE) was assessed after a MSFT. Subjective ratings of mental fatigue were higher after the Stroop task (*p* < 0.001). CP (*p* = 0.0.1), E (*p* < 0.001), vVO_2_max (*p* = 0.020), and estimated VO_2_max (*p* = 0.021) values were negatively affected by mental fatigue. RPE were significantly higher in the mentally fatigued than in the control conditions (*p* = 0.02) post-MSFT. Mental fatigue impairs aerobic and cognitive performance in active male endurance athletes.

## 1. Introduction

Mental fatigue, which has subjective, behavioral and physiological manifestations, is a psychobiological state induced by prolonged periods of demanding cognitive activity [1]. From a subjective standpoint, mental fatigue induces increased feelings of tiredness, lack of energy [2], decreased motivation [3], and alertness [4]. From a behavioral point of view, mental fatigue has been shown to negatively influence performance and cognitive functioning [5,6,7]. Physiologically speaking, mental fatigue may alter brain activity [7,8,9].

Most previous studies that have examined the effects of mental fatigue on physical performance have revealed that mental fatigue does not affect maximal strength, power, and anaerobic work capacity [10,11,12,13]. Only one study reported a decreased leg extension maximal voluntary contraction (796 ± 150 N to 741 ± 137 N) after a 100 min mentally fatiguing task [14]. In contrast, mental fatigue has been consistently shown to decrease endurance performance, as assessed using different tests such as time-clamped self-paced running/cycling protocols and the Yo-Yo intermittent recovery test (decreased time to exhaustion, lowered self-selected power output/velocity, or increased completion time [15]). Nevertheless, these investigations have highlighted the negative effect of mental fatigue in adult team-sport athletes.

It has been shown that younger adults were more affected by mental fatigue tasks than older adults [16]. This difference is nicely mirrored in behavioral, neurophysiological and psychological data. More specifically, younger adult/adolescent participants showed markedly decreased accuracy and motivation, and increased error rates and alpha activity in performing mental fatigue tasks [16]. However, to date, no study has evaluated the effects of mental fatigue on endurance performance in adolescent individual sports participants, in order to better understand whether mental fatigue-related responses in older adults translate to younger adults. For instance, fatigue imposes a clinically relevant burden among athletes. From a clinical standpoint, the present article may allow sports practitioners and coaches to design and adopt *ad hoc* interventions to properly prevent or manage mental fatigue in this particular population.

Evidence reveals that endurance sport performance relies on a complex inter-play of physiological and biomechanical factors [17]. Cardiovascular endurance, which can be defined as “the entire body’s ability to sustain prolonged, dynamic exercise using large muscle groups” ([18], p. 223), is one of the major limiting factors in endurance performance. Indeed, classical measures, such as maximal oxygen uptake (VO_2_max), have been traditionally used in the laboratory or in the field to predict the performance potential of runners [19]. Additionally to the physiological parameters, mental factors would seem to affect cardiovascular endurance performance during running. Previously, McCormick et al. [20] suggested six psychological determinants of endurance performance, namely motivators, mental fatigue, priming interventions, experimenter effects, emotion suppression, and efficacy strength. Typically, mental fatigue has been shown to undermine endurance performance, particularly time to exhaustion [5], running times in a 3 km time trial [21], and performance times in a 5 km running time trial [12], when compared with control conditions in adult athletes. No research, however, has examined the influence of mental fatigue on aerobic performance, assessed using the 20 m multistage fitness test (MSFT), in adolescent active endurance athletes. Therefore, the aim of the present study was to investigate the effects of mental fatigue on cognitive and aerobic performance, assessed using the MSFT and d2 test, respectively, in adolescent active endurance athletes. Specifically, we hypothesized that a 30 min Stroop task leading to mental fatigue would (a) reduce estimated selective attention and estimated VO_2_max performance and (b) increase subjective ratings of mental fatigue and perceived exertion.

## 2. Experimental Section

### 2.1. Participants

Ten adolescent active male endurance athletes (age = 16 ± 1.05 years, height = 1.62 ± 0.04 m, body mass = 55.5 ± 4.2 kg) engaged in middle and long distance track events (i.e., 800, 1500, and 3000 m) volunteered to participate in this study after being informed of the nature and of the possible risks associated with the experiment. The participants were high school team sport athletes. To be eligible to participate in the study, participants were required to meet the following criteria: (a) no consumption of any supplements or drugs; (b) no history of use of medications that could alter the hypothalamic-pituitary-gonadal (HPG) axis, such as anabolic steroids (by responding to the question “Have you used or taken any dietary supplements, drugs or medications in the past 30 days?”); (c) no history of chronic disease, bronchospasm or atopy; (d) no respiratory infection during the previous month (by responding to the question “Were you ill in the past 30 days?”); (e) abstinence from strenuous exercise in the 48 h before testing and (f) not being color blind or vision-impaired (by responding to the questions “Do you have any problems in your vision?”).

Local institutional ethical approval was provided for this study, which was conducted in accordance with the 1964 Helsinki declaration. Written informed assent was obtained from the participants and informed consent from their parents/guardians following verbal description of all experimental details, prior to experimental data collection.

### 2.2. Study Design and Procedure

In this randomized counterbalanced, 2 × 2 cross-over study, reported according to the CONSORT guidelines [22], participants visited the laboratory on three separate occasions at the same time of day (2 p.m.), each separated by 1 week. The participants were familiarized with the testing procedures during visit 1. The order in which participants experienced the two conditions (mentally fatiguing task—experimental condition—and non-mentally fatiguing task—control condition) was randomized in such a way that the effect of the conditions (experimental first—then control versus control first—then experimental) was not confounded with the effect of the order in which the conditions themselves were given (randomized cross-over design). Sample size was *a priori* computed taking into account the repeated measures with one between subjects factor (one half of the subjects undergoing first the mentally fatiguing task and after the non-mentally fatiguing task and the other half of subjects receiving first the non-mentally fatiguing task and thereafter the mentally fatiguing task) and one within subjects factor (all participants undergoing both conditions) design:

(1) $$y_{ijk}=\mu_{k}+y_{ik}+s_{ij}+\varepsilon_{ijk}$$

where $y_{ijk}$ is the response/outcome from the j^th^ participant in the i^th^ sequence under the k^th^ condition, $\mu_{k}$ the effect/outcome of the k^th^ condition, $y_{ik}$ the fixed effect/outcome of the i^th^ sequence under the k condition, $s_{ij}$ the between subjects variability, and, finally, $\varepsilon_{ijk}$ the within subjects variability. With an 80% power, the minimum number of required participants was 8.

The first visit consisted of the collection of anthropometric data. Furthermore, participants were familiarized with rating of perceived exertion (RPE, Borg’s category 6–20 scale) scale [23] as well as the Brunel Mood Scale (BRUMS, [24,25]) for the assessment of mental fatigue. Participants were also familiarized with the mentally fatiguing task (Stroop task), the MSFT and the d2 test. 

During the second and the third visits, participants were asked to provide a subjective rating of mental fatigue using BRUMS, before completing one of two conditions (mentally fatiguing session or non-mentally fatiguing session/control session). In the control session, participants leisurely read from a selection of emotionally neutral magazines for 30 min. In the mentally fatiguing session, participants completed a paper version of the Stroop task. Mental fatigue was again assessed after each condition. After the BRUMS, participants completed the d2 test. After that, they performed a 5–7 min running warm-up and the MSFT. Finally, rating of perceived exertion (RPE) was assessed after a MSFT.

Participants were advised to avoid exercise, caffeine, and alcohol 48 h before each laboratory visit. Food and fluid intake was registered 48 h prior to the first study visit, and subjects were asked to avoid such intake 3 h before the second and the third visits.

#### 2.2.1. Mental Fatigue Task

The Stroop task was used for the mental fatigue task [12]. Four words (red, blue, green, and yellow) were displayed in a random order on five sheets of A4 paper with 45 words printed on each sheet. Participants were required to verbally respond to each word, with the correct response corresponding to the ink color of the word (red, blue, green, and yellow), rather than the words’ meaning. Therefore, if the word “green” was printed in blue ink, the correct response was “blue”. However, if the ink color of the word was red, the correct response corresponded to the meaning of the word, rather than its printed color. Therefore, if the word “green” was displayed in red ink, the correct answer was “green”. Of note, participants repeatedly read the five sheets over and over for 30 min.

A member of the research team recorded the number of incorrect answers with a control sheet, and asked participants to restart the current row of words when the answer was incorrect. Thus, the points awarded for speed and accuracy of responses were noted to increase the motivation of athletes.

#### 2.2.2. Control Task

The control task involved 30 min of reading at a leisurely pace from a selection of magazines, which varied in theme, including sport, cars, and travel. According to the BRUMS [24,25], 30 min of reading from these magazines was emotionally neutral.

#### 2.2.3. Measures

To assess the selective attention of participants, we used the d2-test as developed by Brickenkamp and Oosterveld [26]. The d2-test consists of 14 lines, each containing 47 symbols. A symbol is either a letter p or a letter d with one or two lines (either “or”) above and/or below the letter. The assignment is to mark each letter d that has a total of two lines above and below the letter. In order to make the test perfectly, respondents should not mark any other symbol than a d2, and all d2 symbols have to be marked.

The d2-test is timed, and respondents are given 20 s to complete each line. After these 20 s, respondents have to continue on the next line. The total test lasted 4 min and 40 s. As such, the test assesses concentration in terms of both accuracy and speed.

Two parameters were calculated after completion of the d2 test in our study; concentration performance (CP) and total number of errors made by the participants (E). Concentration performance is assessed as the number of correctly marked d2-symbols minus the number of incorrectly marked symbols (symbols that are not d2-symbols). In addition to concentration performance, we calculated the total number of errors made by the participants (E). The total number of errors is assessed as the number of errors made by failing to identify a correct d2-symbol plus the number of errors made by incorrectly marking symbols that are not d2-symbols.

#### 2.2.4. Aerobic Performance 

The MSFT was conducted as previously described [27]. Briefly, participants ran back and forth between two lines, spaced 20 m apart, in time with the “beep” sounds from an electronic audio recording. Each successful run of the 20 m distance was a completion of a shuttle. The test started with an initial speed of 8 km/h that increased by 0.5 km/h every minute and was stopped if the subject failed to reach the line (within 2 m) for two consecutive ends after a warning. Maximal speed was calculated as the velocity of the last stage fully completed and considered as the speed associated with VO_2_max for the shuttle run test (vVO_2_max). VO_2_max was estimated using the Léger et al. [27] formula. 

#### 2.2.5. Mood

The BRUMS developed by Terry et al. [25] was used to quantify current mood (“How do you feel right now?”) before and after the cognitive tasks. This questionnaire contains 24 items (e.g., “angry, uncertain, miserable, tired, nervous, and energetic”) divided into six respective subscales: anger, confusion, depression, fatigue, tension, and vigor. The items are answered on a 5-point Likert scale (0 = not at all, 1 = a little, 2 = moderately, 3 = quite a bit, and 4 = extremely), and each subscale, with four relevant items, can achieve a raw score in the range from 0 to 16. Only the score for the fatigue subscale was considered in this study as the subjective marker of mental fatigue.

### 2.3. Statistical Analyses

Data were presented as mean values ± standard deviation (SD). Shapiro-Wilk’s test was used to determine the data’s normal distribution. Differences between cases and controls were computed performing repeated measures analysis of variance (ANOVA) analysis, with *post-hoc t*-tests. Pairwise differences between the dependent variables were identified by using paired t-tests, correcting for multiple testing (pre- and post-design, cases versus controls). To allow a better interpretation of the results, the effect sizes (ES) were calculated, using Cohen’s d, correcting for the pre-post design using Morris and DeShon’s equation. A significance level of *p* ≤ 0.05 was used for all analyses. All statistical analyses were carried out using the “Statistical Package for the Social Sciences” for Windows (SPSS Inc., Chicago, IL, USA, version 16.0). Graphs were generated using the commercial software MedCalc Statistical Software (MedCalc Software bvba, Ostend, Belgium; http://www.medcalc.org; 2017; version 17.9.7).

## 3. Results

### 3.1. Selective Attention

There were statistically significant differences in the CP (*p* = 0.001) and errors (*p* < 0.001) values between mentally fatigued and control conditions (Table 1).

### 3.2. Aerobic Performance

There were statistically significant differences (*p* = 0.021; standardized mean difference = 1.13, ES = 1.19 (95% CI 0.81–3.18)) in the estimated VO_2_max values between mentally fatigued and control conditions, with higher estimated VO_2_max values in the control condition than in the mentally fatigued condition. Furthermore, there were higher vVO_2_max values in the control condition than in the mentally fatigued condition (*p* = 0.021; standardized mean difference = 1.13, ES = 1.19, (95% CI 0.86–1.52)) (Table 1).

### 3.3. Subjective Ratings

**Figure 1:**
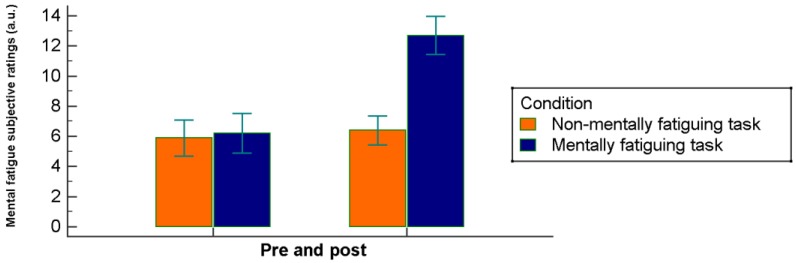
Mental fatigue subjective ratings pre and post broken down to the condition (experimental and control).

Overall, subjective ratings of mental fatigue increased significantly (*F* = 176.40, *p* < 0.0001): in particular, they increased after the mentally fatigued condition (*p* < 0.0001) but not after the control condition (*p* > 0.05). The effect of the condition was, therefore, significant (*F* = 50.91, *p* < 0.0001), as well as the interaction time (pre versus post) × condition (129.60, *p* < 0.0001). Follow-up tests revealed that there were no differences (*p* = 0.600; ES = −0.23 (95% CI −1.48–0.88)) in the pre-cognitive task perception of mental fatigue between mentally fatigued (6.2 ± 1.31) and control conditions (5.9 ± 1.1), but post-condition ratings of mental fatigue were significantly higher in the mentally fatigued condition (12.7 ± 1.2) than in the control condition (6.4 ± 0.9; *p* < 0.001; ES = −5.65 (95% CI −7.34–−5.25)) (Figure 1). 

**Figure 2:**
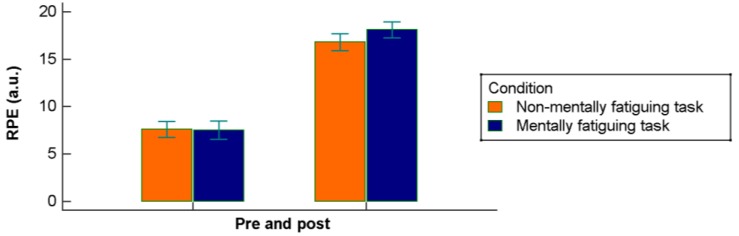
Subjective post-MSFT RPE pre and post broken down to the condition (experimental and control).

Subjective post-MSFT RPE were also higher for the mentally fatigued condition than the control condition (*p* = 0.02; ES = −1.03 (95% CI −2.43–−0.16)) (Table 1, Figure 2).The overall effect of the condition was not significant (*F* = 1.46, *p* = 0.243), whereas the effect of time (pre versus post) and the interaction time × condition were statistically significant (*F* = 1603.80, *p* < 0.00001, and *F* = 8.02, *p* = 0.011, respectively).

## 4. Discussion

To our knowledge, this is the first study that investigated the impact of mental fatigue on selective attention and endurance performance in active male endurance athletes. The findings showed that mental fatigue induced by prolonged periods of a demanding cognitive activity (i.e., 30 min Stroop task) impaired cognitive and aerobic performance, in terms of selective attention and estimated VO_2_max, in active male endurance athletes. Also, subjective ratings of mental fatigue and RPE were higher in the mentally fatigued condition than in the control condition. Therefore, this finding confirms our first hypothesis, and suggests that the Stroop task can be used by strength and conditioning coaches to induce mental fatigue and to avoid mentally fatigued tasks before competition.

### 4.1. Mental Fatigue and Cognitive Performance 

The present study reported that mental fatigue impairs selective attention in term of concentration performance and number of errors. These findings support the results of Boksem et al. [28] and Faber et al. [29], who showed that 2–3 h of mental fatigue task reduces attention performance. More specifically, Faber et al. [29] studied that effect of mental fatigue on selective attention in visual processing by examining differences in processing of task relevant versus task irrelevant information by using Eriksen’s flanker task. In this task, the size of targets and flankers was manipulated to examine the effect of selective attention on the processing of relevant information and irrelevant information, respectively. The authors showed a significant decrease in accuracy and an increase in reaction times after mentally fatiguing tasks. This may be explained by the fact that mental fatigue hampered the suppression of irrelevant information [29]. More specifically, with increasing mental fatigue, the brain activity, elicited by large and small flankers changed gradually from negative to positive, which might reflect a reduction of the suppression of irrelevant information and indicate that our information processing system seems to become less able to block out irrelevant information [29].

### 4.2. Mental Fatigue and Aerobic Performance

The Stroop task requires sustained attention and response inhibition and has been shown to induce a state of mental fatigue [30]. The current findings are in agreement with previous results [5,12,21,31,32], which reported the negative impact of prior mental exertion on endurance performance. In addition, mental fatigue impaired endurance performance during cycling [5] and running exercises [12,21,33]. Previous studies reported that fatigue induced by mental exertion also impairs prolonged intermittent [32] and graded [31] running exercises. Veness et al. [34] investigated the effect of mental fatigue on endurance performance. In this study, ten elite male cricket players performed the Yo-Yo-Intermittent Recovery Level 1 (Yo-Yo-IR1) test with and without engagement in the incongruent Stroop test. The authors reported the reduction of Yo-Yo-IR1 distance covered following the mental fatigue task. Smith et al. [31] reported similar results, and they observed a 15% reduction in the distance covered during the Yo-Yo test. Furthermore, Martin et al. [35] recruited eleven professional and nine recreational road cyclists, who engaged in a modified incongruent color-word Stroop task for 30 min. The authors showed a significant decrease of time trial performance in recreational cyclists only when compared with the control condition, while performance remained unchanged in professional cyclists. These findings may suggest that professional cyclists exhibited stronger inhibitory control than recreational ones [35].

To explain the impairment of endurance performance after mental fatigue, previous studies have suggested that mental fatigue is associated with brain adenosine accumulation [12,36,37,38] that will have a direct impact perception of effort that will in turn impact on endurance performance [39,40].

### 4.3. Mental Fatigue and Subjective Ratings

The relationship between RPE and fatigue is a key principle of the psychobiological model, which is the prevailing framework used to explain the effects of mental fatigue on performance [41]. The model assumes that the level of conscious exertion (i.e., RPE) during an exercise task, which is ostensibly dependent on the degree of corollary discharge, gauges one’s proximity to exhaustion (task disengagement) [5]. Therefore, an increase in RPE theoretically represents a progression towards exhaustion on a physical task, such as the MSFT, with a lower RPE associated with increased time to exhaustion (i.e., improved performance). Accordingly, the present study showed that a lower RPE after the MSFT in the non-mentally fatigued condition compared to the mental fatigue condition was associated with an increase in estimated VO_2_max performance. This finding is in agreement with the results of Penna et al. [42], Smith et al. [31] and Veness et al. [34], who reported a higher RPE during the Yo-Yo test and 1500-m swimming trial in the mental fatigue condition when compared with the control condition.

Interestingly, the association between impairment of endurance performance following completion of the Stroop task and increased perception of effort, could be explained by the similarity in brain areas involved in both mechanisms [41]. Moreover, to explain this result, it is worth noting that prolonged periods of demanding cognitive activity, specifically the Stroop task, is associated with activity in the anterior cingulate cortex [43] and increased cerebral adenosine accumulation which in turn lead to an increased perception of effort during subsequent endurance exercises and impaired motivation (willingness to exert effort, [36]).

The current results are in agreement with previous findings, with subjective ratings of mental fatigue increasing from pre- to post-mentally fatigued task, while remaining unchanged in the control condition [31]. Nevertheless, it is clear that performing the Stroop task used in the current investigation for 30 min successfully induced mental fatigue. Therefore, the general finding is that endurance performance is impaired by mental fatigue and this is possibly mediated by higher than normal perceived exertion and subjective rating of mental fatigue during exercise.

## 5. Limitations of the Study

Some limitations related to the present study have to be noted. First, the major shortcoming is given by the low number of athletes who participated in the present study, which makes it difficult to generalize the findings. Second, the MSFT, which was used to estimate VO_2_max, was applied as an indirect method, which may be considered to be not accurate and precise. However, despite the limitations of this method, it is important to note that this method resembles real situations of practice more closely than other tests.

## 6. Conclusions

The current study demonstrates that mental fatigue impairs whole-body endurance and cognitive performance of active male endurance athletes. Furthermore, perception of effort and subjective rating of mental fatigue decreased after mental fatigue. Practitioners should consider the mental fatigue state of their athletes to optimize endurance performance. Also, they should avoid any tasks requiring sustained attention and causing mental fatigue before competition. Furthermore, sport scientists and coaches should adopt an appropriate strategy, such as pre-competition cognitive strategy or caffeine supplementation to reduce the mental fatigue induced by the high cognitive demands during competition. Moreover, based on the above-mentioned shortcomings, future work is needed to replicate our findings and extend them in fully-powered studies containing both male and female athletes, and, then, within a training paradigm.

## Figures and Tables

**Table 1:** Mean values and standard deviations (SD) of the attention test, aerobic performance variables and rating of perceived exertion (RPE).

Condition	CP	E	vVO_2_max (km/h)	VO_2_max (mL/min/kg)	RPE (a.u.)
MFC (*n* = 10)	71.6 ± 3.1	29.9 ± 3.9	10.2 ± 0.78	33.8 ± 4.7	18.1 ± 1.1
CC	77.2 ± 3.5 *	22.2 ± 3.3 *	11.1 ± 0.8 *	39.2 ± 4.8 *	16.8 ± 1.2 *
ES	1.79 (95% CI 0.41–3.16)	−2.25 (95% CI −3.75–−0.75)	1.19, (95% CI 0.86–1.52)	1.19 (95% CI 0.81–3.18)	−1.03 (95% CI −2.43–−0.16)

* Different from MFC, *p* < 0.05; a.u.: arbitrary units; CC: control condition; CP: concentration performance; E: Errors; ES: effect size; MFC: mentally fatigued condition; RPE.
